# Differential effect of China’s Zero Markup Drug Policy on provider-induced demand in secondary and tertiary hospitals

**DOI:** 10.3389/fpubh.2024.1229722

**Published:** 2024-04-24

**Authors:** Xiaoxi Zhang, Armand Zimmerman, Hongyu Lai, Yanyan Zhang, Zhongyi Tang, Shenglan Tang, Osondu Ogbuoji

**Affiliations:** ^1^School of Global Health, Chinese Center for Tropical Diseases Research, Shanghai Jiao Tong University School of Medicine, Shanghai, China; ^2^Center for Policy Impact in Global Health, Duke Global Health Institute, Duke University, Durham, NC, United States; ^3^School of Data Science, Fudan University, Shanghai, China; ^4^Business School, Hohai University, Nanjing, China; ^5^Shanghai Municipal Health Commission, Shanghai, China; ^6^Duke Department of Population Health Sciences, Duke University, Durham, NC, United States; ^7^Duke Margolis Center for Health Policy, Duke University, Durham, NC, United States

**Keywords:** China, Zero Markup Drug Policy, health policy, health economics, public hospital

## Abstract

Following the marketization of China’s health system in the 1980’s, the government allowed public hospitals to markup the price of certain medications by 15% to compensate for reduced revenue from government subsidies. This incentivized clinicians to induce patient demand for drugs which resulted in higher patient out-of-pocket payments, higher overall medical expenditure, and poor health outcomes. In 2009, China introduced the Zero Markup Drug Policy (ZMDP) which eliminated the 15% markup. Using Shanghai as a case study, this paper analyzes emerging and existing evidence about the impact of ZMDP on hospital expenditure and revenue across secondary and tertiary public hospitals. We use data from 150 public hospitals across Shanghai to examine changes in hospital expenditure and revenue for various health services following the implementation of ZMDP. Our analysis suggests that, across both secondary and tertiary hospitals, the implementation of ZMDP reduced expenditure on drugs but increased expenditure on medical services, exams, and tests thereby increasing hospital revenue and keeping inpatient and outpatient costs unchanged. Moreover, our analysis suggests that tertiary facilities increased their revenue at a faster rate than secondary facilities, likely due to their ability to prescribe more advanced and, therefore, more costly procedures. While rigorous experimental designs are needed to confirm these findings, it appears that ZMDP has not reduced instances of medical expenditure provoked by provider-induced demand (PID) but rather shifted the effect of PID from one revenue source to another with differential effects in secondary vs. tertiary hospitals. Supplemental policies are likely needed to address PID and reduce patient costs.

## Introduction

The marketization of China’s economy since the early 1980’s has led to major challenges and reforms within the health sector. China’s transition to a market economy was followed by the decentralization of its health system. Most notably, the central government relatively rendered the funding of health care the responsibility of local authorities and subsequently reduced its own investments in health care services (1). The government’s share of total health expenditure, for example, dropped from 32.16 to 15.84% from 1978 to 1999 (2). In addition, government subsidies comprised more than 60% of total revenue across public hospitals, prior to the reform in the early 1980s, but less than 25% in 2008 (3). As a result of the decline in government support, public hospitals increasingly relied on profits from the sale of pharmaceuticals and services to cover expenses (4, 5). Under this marketized health system, the government limited the cost of basic health services to keep health care affordable to patients but also allowed public health facilities to markup the cost of prescribed drugs by 15% and traditional Chinese medicines by 20–25% to compensate for reduced revenue from other prescribed services and government subsidies (6).

The government sanctioned 15% markup on the purchase price of prescribed drugs, in addition to a markup on the price of certain diagnostic procedures and a reduction in the price of basic health services, altered public hospital behavior. Now operating under a for-profit system, public hospitals began overprescribing healthcare services to meet profit margins, thereby increasing patient costs (7). By 2000, out-of-pocket payments comprised 60% of total health expenditure in China compared to only 20% in 1980 (8). Moreover, overprescribing healthcare services, notably antibiotics and hormones, raised concerns over the threat of antimicrobial resistance, severe adverse events, and the erosion of patient trust in physicians (9, 10). Aware of these undesirable outcomes, China’s government began a series of health reforms in 2009, intending to improve the quality of care at public hospitals and achieve affordable basic healthcare services for all (11).

A key component of the health sector reforms that China began implementing in 2009 is the Zero Markup Drug Policy (ZMDP). ZMDP aims to establish a sustainable compensation mechanism within public hospitals that removes economic incentives for overprescribing. Specifically, ZMDP aims to achieve this goal by eliminating markups between wholesale and retail prices of essential medicines. Other reforms implemented alongside ZMDP include policies that allocate government subsidies to public hospitals to compensate for reduced profits from drug prescriptions, transition public hospital healthcare provider salaries from a fee-for-service to a performance-based payment scheme, and increase the price of different health services to account for economic development and inflation in China over previous decades (12). Since 2009, ZMDP has been implemented with varying roll-out strategies across numerous provinces in China.

In Shanghai, ZMDP was implemented from 2015 to 2017 in public hospitals using a stepwise approach to eliminate markups on essential medicine prices. Specifically, the allowable markup on essential medicine prices was reduced from 15 to 10% in 2015, 10 to 5% in 2016, and 5 to 0% in 2017 (13). By 2017, all public hospitals in Shanghai had fully implemented ZMDP. Using Shanghai as a case study, this paper aims to collate emerging and existing evidence about the impact of ZMDP on provider-induced demand across secondary and tertiary public hospitals. We use data (derived from the Shanghai Municipal Health Commission) from 150 public hospitals to examine changes in hospital expenditure and revenue for different health services from 2015 to 2019. We compare these results with previous studies evaluating the effectiveness of ZMDP, and use our findings to discuss the impact that ZMDP has likely had on the frequency of provider-induced demand in public hospitals.

## Overview of provider-induced demand

Provider induced demand (PID) occurs when a physician (or other healthcare provider) “influences a patient’s demand for care against the physician’s [or other healthcare provider’s] interpretation of the best interests of the patient” (14). Conceptually, if a provider demands a service that a patient would not have demanded if he or she had the same information as the provider, then the provider has not acted as a perfect agent of the patient and therefore PID has occurred (15). Factors contributing to PID may include the provider-to-patient population ratio, price of medical services, provider compensation scheme, size of the health facility, the patient’s clinical and socioeconomic characteristics, and the patient’s health insurance coverage (16).

Whether or not one or multiple of these factors underlies a given instance of PID is circumstantial and requires consideration of the health system in which that instance of PID occurred. For example, an increase in the provider-to-patient population ratio decreases the market share of each physician in the population. In a market where the prices of healthcare services are fixed, physicians may react to an increase in the provider-to-patient population ratio by inducing demand for their services to maintain or increase their income (17). Similarly, a reduction in the price of medical services in a market where prices are fixed may prompt physicians to induce demand to increase service utilization and therefore their income (18). As another example, larger hospitals typically have higher fixed costs. Large hospitals may therefore induce demand for expensive medical services to maintain high returns on investment (19).

In the context of China’s post-1978 public health system, as described above, occurrences of PID were largely driven by physician compensation schemes and medical service prices. Merit pay, for example, was the largest component of a physician’s income in most of the large hospitals in China and was based on the total profit generated by the clinical departments of the hospital where physicians worked (20). With a government authorized 15% markup on essential medicine prices (as well as other high-technology diagnostic service prices), and a fee-for-service based income, physicians were incentivized to prioritize drug and diagnostic prescriptions over basic primary care services. In 2012, 40% of the total revenue generated by public hospitals in China came from drug sales while 50 and 40% of patient expenditure per outpatient and inpatient visit, respectively, was on drugs (21). A primary aim of ZMDP is to reduce public hospital reliance on drug sales. In this review, we examine changes in public hospital expenditure and revenue across secondary and tertiary facilities during the period following implementation of ZMDP in Shanghai to determine if ZMDP decreased reliance on drug sales and therefore decreased instances of PID.

## Overview of secondary and tertiary hospitals

In China, secondary and tertiary hospitals differ primarily in terms of the population served, services provided, and certifications required (22, 23). Secondary hospitals typically serve nearby communities and provide basic medical services, while tertiary hospitals serve larger catchment areas spanning whole cities or provinces and provide specialized medical services, advanced teaching, and scientific research. In addition, government regulations define hospitals as secondary or tertiary based on a set of indicators that consider the number of beds, staff, departments, and fixed assets available, the type of medical equipment used, the quality of management, and the quality of healthcare services provided. For example, secondary hospitals must have between 100 and 499 inpatient beds, there must be at least 0.88 health technicians and 0.4 nurses per inpatient bed, and each hospital department must have at least one attending physician or a physician of higher seniority. In contrast, tertiary hospitals must have 500 or more inpatient beds, there must be at least 1.03 health technicians and 0.4 nurses per inpatient bed, and each hospital department must have at least one associate physician or physician of higher seniority.

## Summary of existing evidence on the effectiveness of ZMDP

Numerous studies have examined the impact of ZMDP on public hospital expenditure and revenue in different regions of China. Using a difference-in-differences approach, Zhang et al. (24) compared public hospital expenditure across two counties in Hubei province (an intervention county where ZMDP was piloted and a control county where ZDMP was not piloted) using data from 16,721 inpatient admissions between 2011 and 2013. The authors found that while per patient drug expenditure decreased relative to the control county, total per patient expenditure and out-of-pocket per patient expenditure increased (24). Using a similar methodology, Fu et al. (25) compared public hospital expenditure across 187 hospitals in Fujian province (22 intervention hospitals where ZDMP was implemented and 165 control hospitals where ZMDP was not implemented) between 2008 and 2014. The authors found that average drug expenditure per inpatient and outpatient as well as total expenditure per inpatient and outpatient were lower across hospitals where ZDMP was implemented relative to the control group (25). Ni et al. (26) also used a difference-in-differences approach to compare expenditure across 34 public hospitals in Shanxi province (22 intervention hospitals where ZDMP was implemented and 12 control hospitals where ZDMP was not implemented) between 2015 and 2017. Again, the authors found that the introduction of ZMDP significantly reduced per inpatient drug expenditure relative to the control group, but also increased per inpatient diagnostic, treatment, material, and services expenditure (26). Fu et al. (27) evaluated changes in public hospital expenditure across 1,880 counties in China that introduced ZMDP between 2009 and 2014, and found that ZMDP reduced per patient drug expenditure but increased per patient medical service, diagnostic, and consumable expenditure with no significant change in total per patient expenditure. Zhang et al. (28) compared expenditure before and after the implementation of ZMDP in 130 hospitals across Zhejiang province and found that ZMDP decreased per patient drug expenditure and increased per patient service expenditure. Lastly, a systematic review conducted in 2021, which includes 15 studies evaluating the impact of ZMDP on public hospital expenditure, concluded that in most cases ZMDP reduced per patient drug expenditure with varying effects on other medical expenditure and service utilization (29).

Few studies have examined the differential impact of ZMDP on secondary and tertiary public hospital expenditure and revenue. Li et al. (30) analyzed data from 2013 to 2018 for 658 secondary and tertiary hospitals across central China. The authors found that drug revenue as a proportion of total revenue decreased more substantially among tertiary hospitals in comparison to secondary hospitals following the implementation of ZMDP (30). Moreover, the authors explain this observation by arguing that tertiary hospitals offer a larger and more advanced portfolio of medical services and therefore have more channels through which to generate income relative to secondary hospitals. Nonetheless, additional evidence is needed to better understand the differential impact of ZMDP on secondary and tertiary hospitals.

## Trends in drug expenditure and revenue: emerging evidence

We used data from 150 public hospitals across Shanghai, between 2015 and 2019, to examine changes in mean per patient drug, medical service, and diagnostic expenditure and revenue among secondary and tertiary facilities following the implementation of ZMDP. The data was retrieved from the Shanghai Municipal Health Commission. The data we received was disaggregated to the level of individual hospitals. As such, annual patient volume, drug expenditure, medical service expenditure, and diagnostic expenditure for each hospital was available in the dataset. We divided expenditures by patient volume to get expenditure per patient for each hospital and took the average across all hospitals in the dataset to get mean per patient expenditure. Using 2015 as the baseline year (the year ZMDP was implemented), we report percent changes in per patient expenditure over the baseline for 2016, 2017, 2018, and 2019.

**Figure 1:**
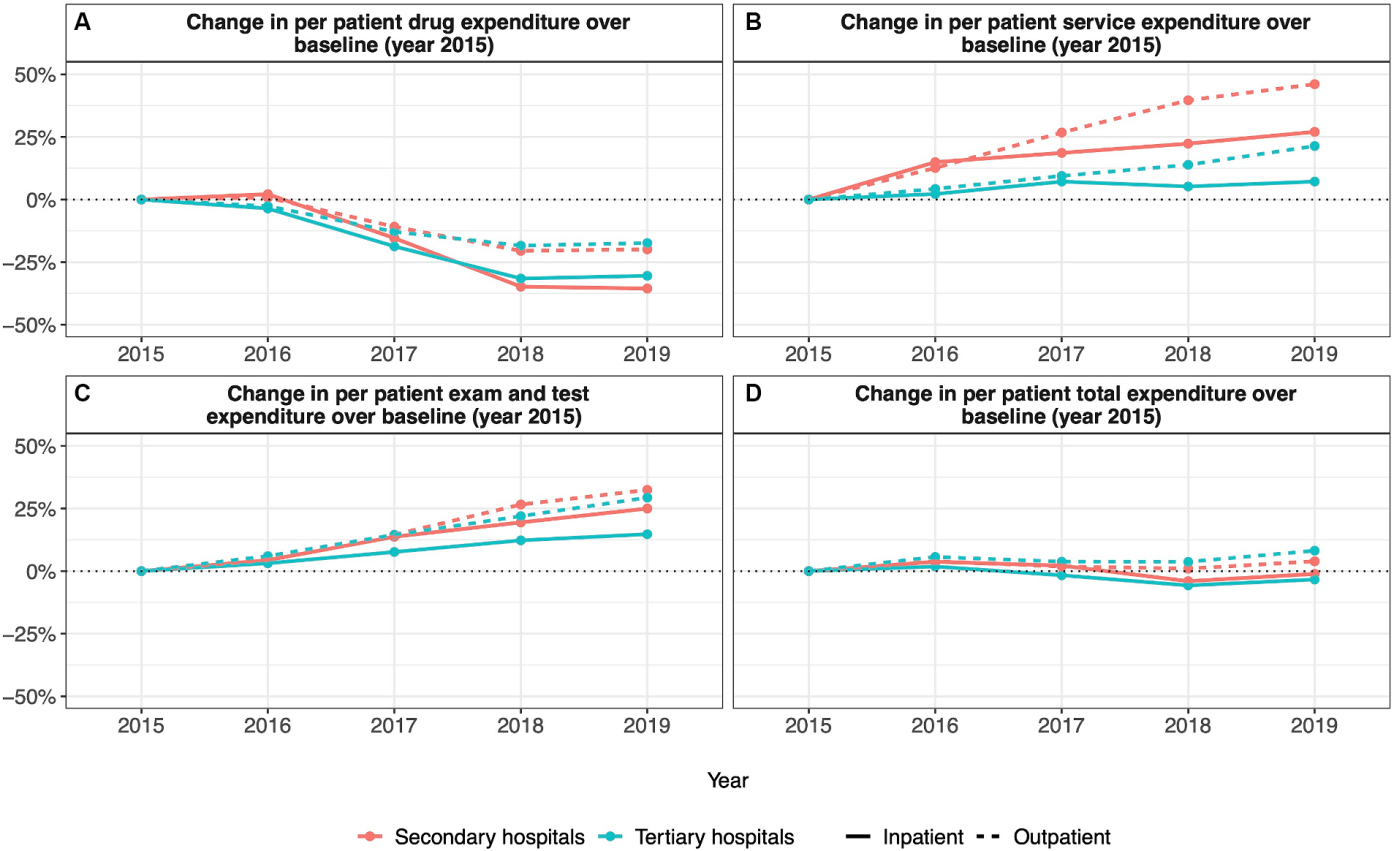
Annual percentage change in per patient hospital expenditures relative to 2015 **(A)** change in per patient drug expenditure; **(B)** change in per patient service expenditure; **(C)** change in per patient exam and test expenditure; **(D)** change in per patient total expenditure).

**Figure 2:**
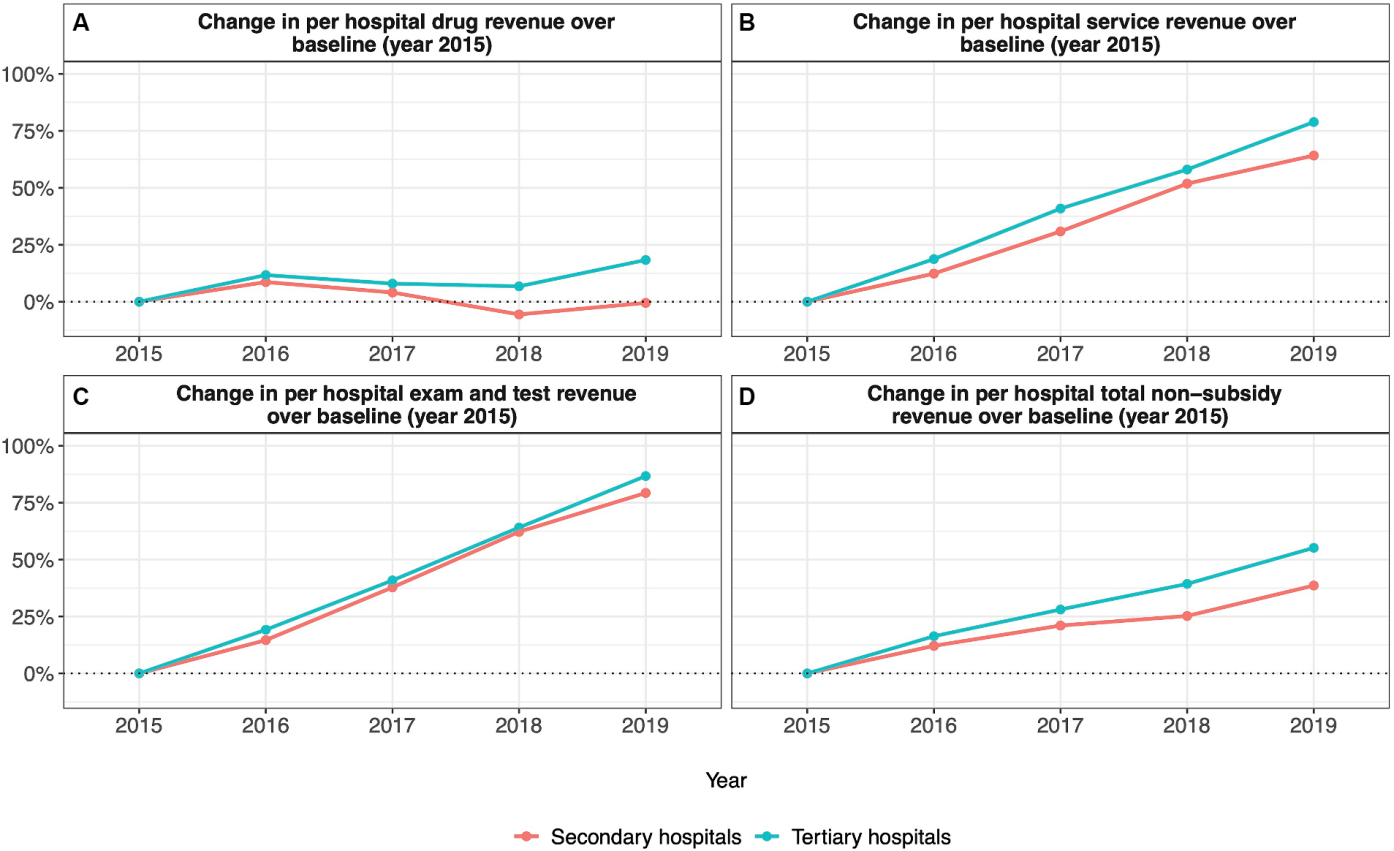
Annual percentage change in per hospital revenue relative to 2015 **(A)** change in per hospital drug revenue; **(B)** change in per hospital service revenue; **(C)** change in per hospital exam and test revenue; **(D)** change in per hospital total nonsubsidy revenue).

Among secondary hospitals, per-inpatient drug expenditure increased over baseline by 2.15% in 2016 and subsequently declined to reach 35.54% below baseline in 2019 (Figure 1A). Similarly, per outpatient drug expenditure increased over baseline by 0.76% in 2016 and then declined to 20.49 and 19.87% below baseline in years 2018 and 2019, respectively (Figure 1A). Despite overall reductions in per-inpatient and outpatient drug expenditure, per-hospital drug revenue fluctuated from 8.63% over baseline in 2016 to a minimum of 5.58% below baseline in 2018 (Figure 2A).

Among tertiary hospitals, per-inpatient drug expenditure fell below baseline values in all years after 2015, reaching a maximum reduction of 31.53% below baseline in 2018 (Figure 1A). Per outpatient drug expenditure followed the same trend, reaching a maximum reduction of 18.41% below the baseline in 2018 (Figure 1A). Again, despite reductions in per-inpatient and outpatient drug expenditure, per-hospital drug revenue was higher than baseline values for all years after 2015, reaching a maximum of 18.32% above baseline in 2019 (Figure 2A).

## Trends in medical service expenditure and revenue: emerging evidence

Among secondary hospitals, per-inpatient service expenditure was above baseline values in all years after 2015, reaching a maximum increase of 27.04% above baseline in 2019 (Figure 1B). Per outpatient service expenditure followed the same trend, reaching a maximum increase of 46.09% above baseline in 2019 (Figure 1B). Per hospital service revenue also remained above baseline values in all years after 2015, reaching a maximum increase of 64.20% above baseline in 2019 (Figure 2B).

Among tertiary hospitals, per-inpatient service expenditure was above baseline values in all years after 2015, reaching a maximum increase of 7.18% above baseline in 2019 (Figure 1B). Per outpatient service expenditure followed the same trend, reaching a maximum of 21.37% above baseline 2019 (Figure 1B). Per hospital service revenue also remained above baseline values in all years after 2015, reaching a maximum increase of 78.89% above baseline in 2019 (Figure 2B).

## Trends in exam and test expenditure and revenue: emerging evidence

Among secondary hospitals, per inpatient exam and test expenditure was above baseline values from 2016 to 2019, reaching a maximum increase of 25.00% above baseline in 2019 (Figure 1C). Per outpatient exam and test expenditure followed the same trend, reaching a maximum increase of 32.45% above baseline in 2019 (Figure 1C). Per hospital exam and test revenue also increased above baseline values from 2016 to 2019, reaching a maximum increase of 79.27% above baseline in 2019 (Figure 2C).

Among tertiary hospitals, per inpatient exam and test expenditure was above baseline values from 2016 to 2019, reaching a maximum increase of 14.73% above baseline in 2019 (Figure 1C). Per outpatient exam and test expenditure followed the same trend, reaching a maximum increase of 29.36% above baseline in 2019 (Figure 1C). Per hospital exam and test revenue also increased above baseline values from 2016 to 2019, reaching a maximum increase of 86.69% above baseline in 2019 (Figure 2C).

## Trends in total expenditure and revenue: emerging evidence

Among secondary hospitals, total per-inpatient expenditure remained unchanged from 2016 to 2019, fluctuating by no more than 4.00% above or below baseline values (Figure 1D). Total per outpatient expenditure followed a similar trend decreasing by no more than 4.00% below baseline from 2016 to 2019 (Figure 1D). Total per hospital non-subsidy revenue, however, was above baseline values from 2016 to 2019, reaching a maximum increase of 38.61% above baseline in 2019 (Figure 2D). Total per hospital subsidy revenue was also above baseline values from 2016 to 2019, reaching a maximum increase of 80.00% above baseline in 2019 (Table 1).

**Table 1:** Mean annual subsidy revenue per hospital.

		2015	2016	2017	2018	2019
Secondary hospitals	Subsidy revenue (100 million CNY)	0.60	0.74	0.94	0.95	1.08
	Percent change	Reference	23.33%	56.67%	58.33%	80.00%
Tertiary hospitals	Subsidy revenue (100 million CNY)	1.05	1.26	1.56	1.58	2.50
	Percent change	Reference	20.00%	48.57%	50.48%	138.10%

Among tertiary hospitals, total per inpatient expenditure increased to 1.83% above baseline in 2016 and subsequently decreased to 5.72 and 3.36% below baseline by 2018 and 2019, respectively (Figure 1D). Total per outpatient expenditure was above baseline values from 2016 to 2019, reaching a maximum increase of 8.16% above baseline in 2019 (Figure 1D). Total per hospital non-subsidy revenue was above baseline values from 2016 to 2019, reaching a maximum increase of 55.17% above baseline in 2019 (Figure 2D). Total per hospital subsidy revenue was also above baseline values from 2016 to 2019, reaching a maximum increase of 138.10% above baseline in 2019 (Table 1).

## Trends in patient volume: emerging evidence

Among secondary hospitals, annual inpatient volume was above baseline values from 2016 to 2019, increasing to a maximum of 28.26% above baseline in 2019 (Table 2). Annual outpatient volume followed the same trend, reaching a maximum of 4.77% above baseline in 2019 (Table 2).

**Table 2:** Mean annual patient volume per hospital.

			2015	2016	2017	2018	2019
Secondary hospitals	Inpatient	*N* (hundreds)	153.80	165.91	175.35	184.67	197.27
		Percent change	Reference	7.87%	14.01%	20.07%	28.26%
	Outpatient	*N* (hundreds)	8104.03	8181.76	8151.41	8221.28	8490.38
		Percent change	Reference	0.96%	0.58%	1.45%	4.77%
Tertiary hospitals	Inpatient	*N* (hundreds)	519.61	588.69	634.63	684.67	742.03
		Percent change	Reference	13.30%	22.14%	31.77%	42.81%
	Outpatient	*N* (hundreds)	22361.36	23566.534	23869.05	24603.27	25539.50
		Percent change	Reference	5.39%	6.74%	10.03%	14.21%

Among tertiary hospitals, annual inpatient volume was above baseline values from 2016 to 2019, increasing to a maximum of 42.81% above baseline in 2019 (Table 2). Annual outpatient volume was also above baseline values from 2016 to 2019, increasing to a maximum of 14.21% above baseline in 2019 (Table 2).

## Discussion

**Figure 3:**
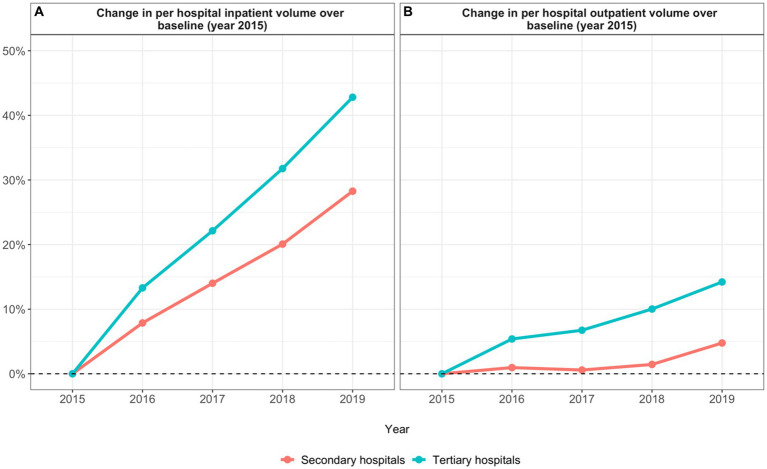
Annual percentage change in hospital patient volume **(A)** change in per hospital inpatient volume; **(B)** change in per hospital outpatient volume).

The evidence presented in this review has important implications regarding the extent to which ZMDP achieved its intended goals. First, ZMDP effectively reduced drug expenditure. Previous evidence presented above shows that, in hospitals across China where ZMDP was introduced, drug expenditure declined in the years following implementation of the policy (22–27). In addition, our analysis of Shanghai data further shows that both per inpatient and per outpatient drug expenditure in secondary and tertiary hospitals declined following the implementation of ZMDP. Thus, it appears ZMDP reduced PID for drugs and shifted hospitals away from an overreliance on drug prescriptions as a primary revenue stream. Interestingly, however, despite reductions in per patient drug expenditure, tertiary hospitals in our dataset increased drug revenue in the years after ZMDP implementation. This increase may be explained by increases in annual patient volume as seen in Figures 3A,B. These observed increases in annual patient volume may be driven by increases in the average income level of Chinese residents which has driven an increase in the demand for medical care (31). Furthermore, larger increases in annual patient volume among tertiary hospitals in comparison to secondary hospitals suggest a growing demand for specialized medical care.

ZMDP also effectively increased medical service expenditure. Previous evidence shows that in various regions of China, hospitals that implemented ZMDP reported higher rates of medical service expenditure in subsequent years (24–27). Our analysis of data from Shanghai also shows that per inpatient and per outpatient medical service expenditure increased across both secondary and tertiary hospitals in the years following the implementation of ZMDP. Notably, however, annual percent increases in medical service revenue were higher in tertiary hospitals even though annual percent increases in per patient medical service expenditure were higher in secondary hospitals. These results suggest that ZMDP, and its accompanying policies that raised the price of medical services, achieved the goal of increasing hospital revenue from medical service fees. Furthermore, tertiary hospitals were more effective than secondary hospitals in increasing medical service revenue, possibly due to both a larger increase in annual patient volume among tertiary hospitals than secondary hospitals (Figures 3A,B) and a greater need for medical services among the more complicated and severe cases that tertiary hospitals serve and therefore greater use of such services. However, it is also important to note that total health expenditure and total hospital expenditure in Shanghai has been increasing since 2002, so it is difficult to ascertain whether or not the increases in medical service revenue since 2015 can be attributed primarily to ZMDP (32).

ZMDP increased exam and test expenditure as well. Again, previous evidence shows that in different regions across China hospital exam and test expenditure increased after the implementation of ZMDP (24–27). Our analysis confirms this finding and shows that per inpatient and per outpatient exam and test expenditure increased in both secondary and tertiary hospitals in the years following ZMDP implementation. In addition, our analysis shows that annual percent increases in exam and test revenue were higher among tertiary hospitals compared to secondary hospitals even though annual percent increases in per patient exam and test expenditure were higher among secondary hospitals. These results suggest that ZMDP failed to control examination, equipment, and testing costs. In both secondary and tertiary hospitals, providers likely induced demand for exam and test services following the implementation of ZMDP to make up for lost revenue from drug prescriptions. However, tertiary hospitals likely experienced larger gains in exam and test revenue in comparison to secondary hospitals because these facilities saw a larger increase in annual patient volume and are also equipped with more advanced diagnostic equipment and can therefore provide more expensive exam and test services (33). Again, total health expenditure and total hospital expenditure in Shanghai have been increasing since 2002 so it is difficult to ascribe the increase in exam and test revenue to ZMDP alone.

**Figure 4:**
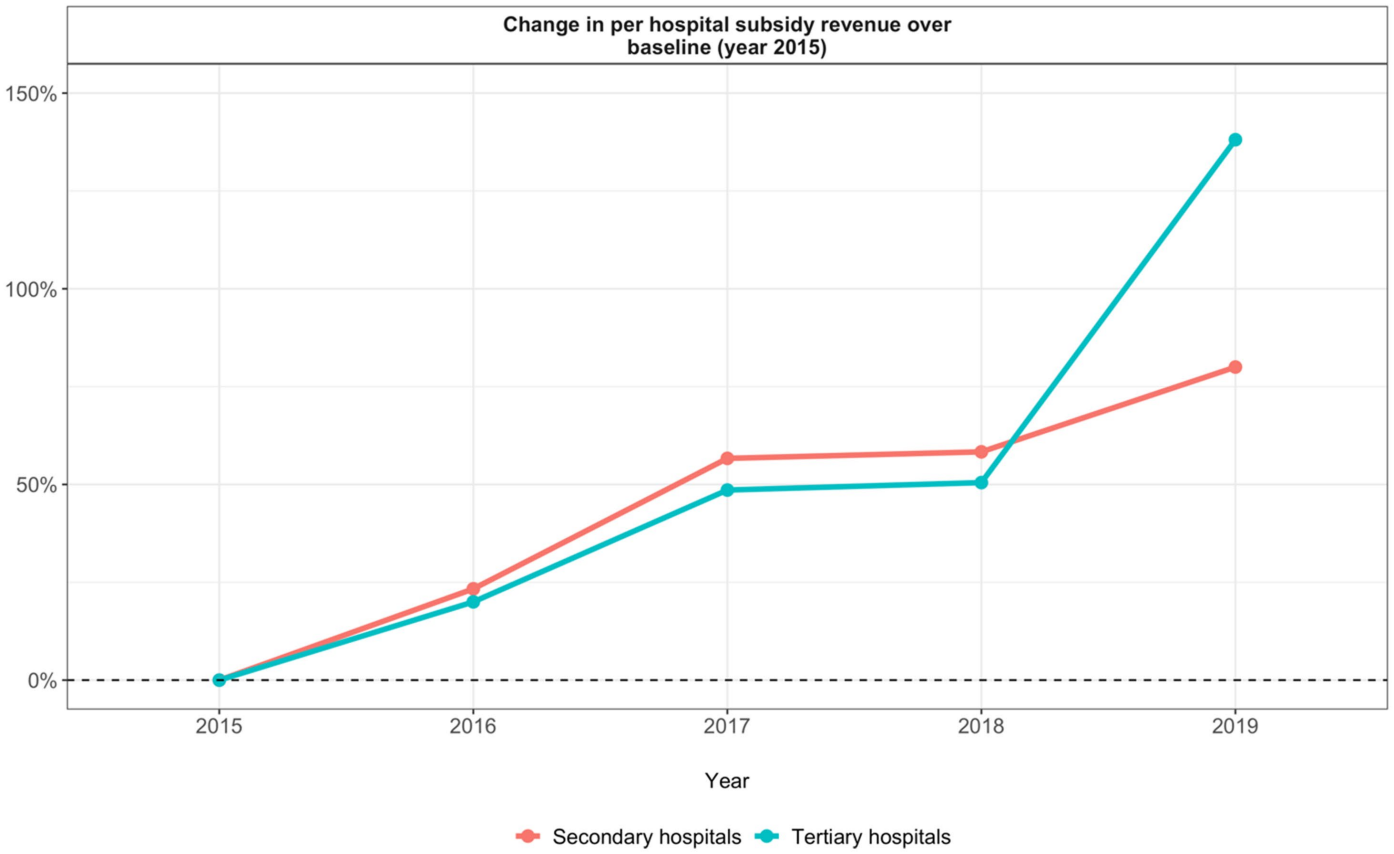
Annual percentage change in hospital subsidy revenue.

Ultimately, additional policies are needed to ensure ZMDP makes healthcare more affordable to patients. Our analysis shows that, across both secondary and tertiary hospitals, average per hospital non-subsidy revenue increased after ZMDP implementation while average per inpatient and per outpatient total expenditure remained constant (within the per-patient total expenditure: drug expenditure decreased, medical service expenditure increased, and exam and test expenditure increased). These findings suggest that ZMDP incentivized providers to induce demand for medical services and exams/tests to make up for reductions in revenue from drug prescriptions thereby increasing hospital revenue and keeping total patient costs unchanged. Other studies also conclude that ZMDP did not significantly reduce out-of-pocket costs to patients (22, 34, 35). In light of these findings, policymakers should consider subsequent reforms of healthcare provider payment mechanisms to ensure that provider income is not reliant on the quantity of medicines or services provided (36). It is also important to note that tertiary hospitals were able to increase their total revenue at a faster rate than secondary hospitals and benefited from higher annual percent increases in government subsidies (Figure 4). Other policies to consider may therefore be reforms to subsidy allocation that ensure secondary hospitals have sufficient funding to offset reduced drug revenue.

## Conclusion

Overall, ZMDP has successfully altered the revenue composition of public hospitals across China. Specifically, the policy has made public hospitals less reliant on revenue from drug prescriptions and more reliant on the provision of medical services and exams/tests. Thus, it appears that the implementation of ZMDP has not reduced instances of medical expenditure provoked by provider-induced demand (PID) but rather shifted the effect of PID from one revenue source to another with differential effects in secondary vs. tertiary hospitals. To ensure reductions in drug prices translate to reductions in out-of-pocket expenses for patients, ZMDP should be supplemented with policies to reform provider compensation mechanisms, control exam and test prices, and ensure secondary hospitals have sufficient subsidies to counterbalance lost drug revenue without increasing costs to the patient. It is important to note that the emerging evidence presented in this review is observational and does not show a causal relationship between the implementation of ZMDP and subsequent changes in hospital expenditure and revenue. Additional studies with rigorous experimental or quasi experimental designs are needed to confirm the direction and magnitude of ZMDP’s impact on hospital expenditure, revenue, patient volume, and PID in secondary and tertiary hospitals.

## Data availability statement

The original contributions presented in the study are included in the article/supplementary materials, further inquiries can be directed to the corresponding author.

## Author contributions

XZ, AZ, ST, and OO conceptualized the paper. AZ, XZ, ST, and OO developed the methodology. XZ, AZ, HL, and YZ conducted the analysis and created visualizations. ZT curated the data. AZ wrote the original draft. XZ, AZ, ST, and OO reviewed and edited the draft. ST and OO provided supervision. XZ acquired funding. All authors contributed to the article and approved the submitted version.
